# Functional links between the microbiome and the molecular pathways of colorectal carcinogenesis

**DOI:** 10.1007/s10555-024-10215-5

**Published:** 2024-09-28

**Authors:** Jessica Permain, Barry Hock, Timothy Eglinton, Rachel Purcell

**Affiliations:** 1 Department of Surgery and Critical Care, University of Otago, Christchurch, New Zealand; 2 Department of Pathology and Biomedical Science, University of Otago, Christchurch, New Zealand

**Keywords:** Colorectal cancer, Tumour microenvironment, Molecular pathways

## Abstract

Colorectal cancer (CRC) is a common cancer, with a concerning rise in early-onset CRC cases, signalling a shift in disease epidemiology. Whilst our understanding of the molecular underpinnings of CRC has expanded, the complexities underlying its initiation remain elusive, with emerging evidence implicating the microbiome in CRC pathogenesis. This review synthesizes current knowledge on the intricate interplay between the microbiome, tumour microenvironment (TME), and molecular pathways driving CRC carcinogenesis. Recent studies have reported how the microbiome may modulate the TME and tumour immune responses, consequently influencing cancer progression, and whilst specific bacteria have been linked with CRC, the underlying mechanisms remains poorly understood. By elucidating the functional links between microbial landscapes and carcinogenesis pathways, this review offers insights into how bacteria orchestrate diverse pathways of CRC development, shedding light on potential therapeutic targets and personalized intervention strategies.

## Introduction

Colorectal cancer (CRC) is one of the most common cancers, with a global cancer incidence rate ranging from less than 5/100,000 in some African nations to more than 40/100,000 in some countries in North America, Europe and Oceania [1, 2]. Whilst the overall incidence rate is expected to remain the same for the next twenty years, the incidence of sporadic disease in younger individuals < 50 years of age is expected to increase. Early-onset CRC has already increased 63% between 1988 and 2015 and is expected to further double by 2030 [3]. Such a rapid increase suggests that environmental factors impact carcinogenesis and has focused attention on the role of the microbiome in the CRC pathogenesis.

Our knowledge of the molecular pathways leading to CRC carcinogenesis has expanded in recent decades; however, the initiating molecular changes leading to carcinogenesis are complex and poorly understood, with many factors, including the microbiome, influencing how an individual develops CRC [4, 5]. Recent research has focused on how the microbiome interacts with the tumour microenvironment (TME), including the immune system, both directly and systemically, often resulting in activation of various immune responses which, in turn, alter the progression of carcinogenesis. Many studies have identified strong associations between specific bacteria, molecular characteristics and patient outcomes. However, whilst associations between specific bacteria and CRC progression have been well documented, it is critical to understand the underlying mechanisms of the bacteria involved. Both the gut and the tumour microbiomes influence the molecular characteristics, immune profile and tumour microenvironment of CRC, which in turn alter pathways of carcinogenesis and subtypes to which the tumours are classified. This review aims to describe the functional links between the tumour-resident microbial landscape, the gut microbiome and the molecular pathways of carcinogenesis to better understand how bacteria influence different pathways of CRC development.

## Molecular pathways of CRC development

**Fig. 1:**
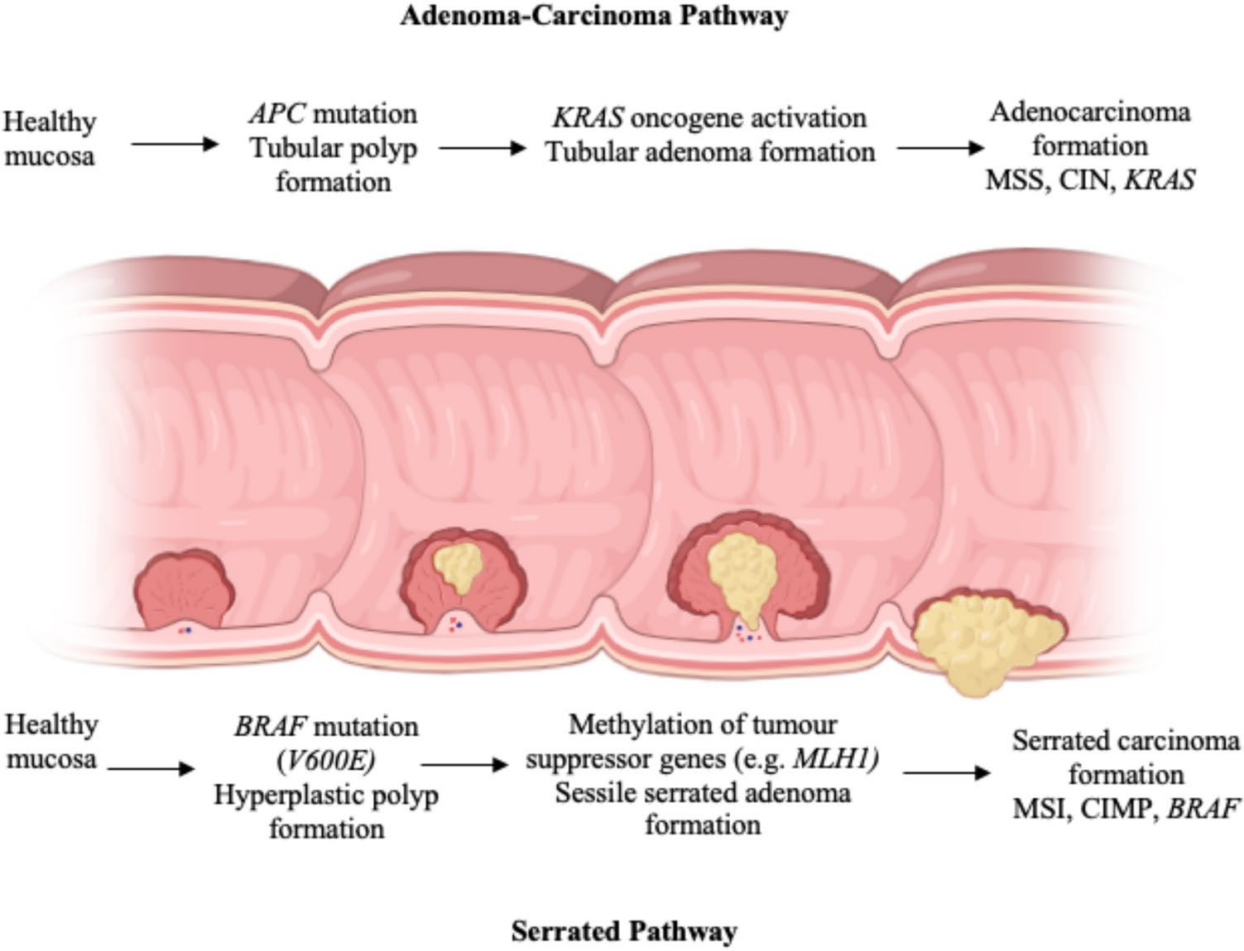
Pathways of colorectal carcinogenesis. Image created with Biorender[83] © 2022, adapted from Keum and Giovannucci 2019

The two most common pathways of colorectal carcinogenesis are the adenoma-carcinoma pathway and the serrated pathway [6]. The adenoma-carcinoma pathway (Fig. 1) accounts for approximately 70% of CRC and is typically associated with the early acquisition of *APC* mutations, which are reported in 70–80% of resulting adenocarcinomas [7, 8]. Downstream acquisition of chromosomal instability (CIN) occurs, followed by *KRAS* mutations, which occur in 20–38% of sporadic colorectal cancer [9]. In addition, *MYC* and *WNT* activation occurs, leading to increased cellular proliferation and oncogenic potential (Fig. 1) [10, 11]. Conventional adenomas and carcinomas arising from this pathway are more frequently found in the left side of the colon and patients tend to have a lower 5-year survival rate, compared to those with cancers arising from the serrated pathway [12].

Tumours arising from the serrated pathway are thought to arise from sessile serrated adenomas and account for approximately 15% of all sporadic CRC cases. Very rarely are APC mutations found in CRC arising from the serrated pathway, with a recent study reporting that truncating *APC* mutations occur in only 8% of serrated CRC, compared to the 70–80% of adenoma-carcinoma cases [22]. Precursor lesions are characterised by a serrated “saw-tooth” like appearance. Tumours frequently express *BRAF* mutations (> 80%), specifically the *V600E* mutation (Fig. 1) [15], which has an activating missense mutation in codon 600 of exon 15 of the *BRAF* gene, leading to constitutive activation of the MAPK pathway, resulting in increased cellular proliferation and decreased apoptosis [15]. The serrated pathway is often associated with microsatellite instability (MSI) [16] and/or a CpG island methylator (CIMP) phenotype, which occurs in approximately 30–40% of CRC and 79% of MSI-positive CRC [17–19] [20, 21]. This hypermethylation typically causes methylation of tumour suppressor genes, such as *MLH1,* resulting in tumour formation [21].

Recurrence patterns differ between cancers arising from each pathway, with microsatellite stable (MSS) cancers arising from the adenoma-carcinoma pathway more likely to be associated with lung and liver metastasis, compared to high microsatellite instability (MSI-H) cancers arising from the serrated pathway, which tend to be associated with peritoneal metastases [13]. *KRAS* mutations are found in 40% of metastatic colorectal cancer, whilst BRAF mutations are only seen in 10–15% of metastatic disease [14], suggesting that the adenoma-carcinoma pathway has a greater metastatic potential.

There remains significant heterogeneity within these complex pathways with considerable overlap of both molecular and clinicopathological characteristics. This limits their utility in prognostication and targeted treatment and also when defining functional links to pathogenesis. The consensus molecular subtype (CMS) classification was introduced in 2015 in an effort to overcome these drawbacks and provide a more robust and universal classification system. The CMS classification was developed using 18 different CRC genomic datasets, containing data from 4151 patients, to which six expert groups applied a subtyping classification algorithm, and an association network was developed to find consensus [23]. The end result was four subtypes: broadly speaking, CMS1 relates to the serrated pathway, and CMS2-4 relates to subtypes of the CIN pathway, whilst allowing more detailed characterisation of the molecular genetics. In this review, bacterial associations and functional links with both CMS and traditional molecular pathways are described, including their interaction with the TME and immune system.

## Bacterial associations and functional links with the CMS subtypes of colorectal cancer

### Consensus molecular subtype 1

**Fig. 2:**
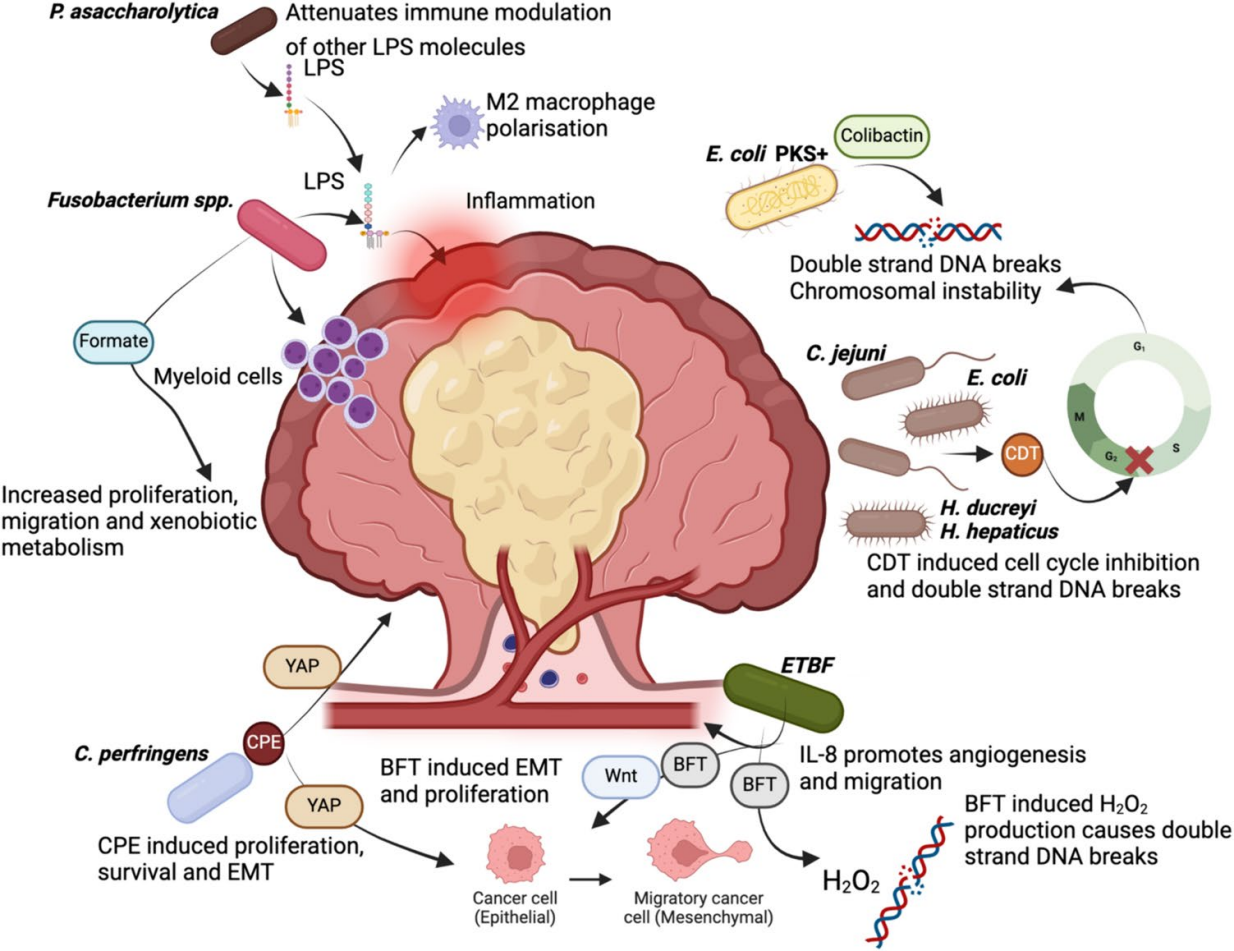
Bacterial mechanisms promoting colorectal carcinogenesis. Bacteria and their known mechanisms of promoting colorectal cancer within the tumour microenvironment. *Fusobacterium* spp. induce myeloid cell infiltration; LPS causes M2 macrophage polarisation and induces a proinflammatory TME promoting CMS1 type tumour development. Formate, a metabolite of *Fusobacterium* spp., induces AhR signalling to promote cellular proliferation and migration. *P. asaccharolytica* LPS attenuates the immunomodulatory effects of other LPS molecules promoting CMS1 characteristics. *E. coli PKS* + via colibactin, induces double strand DNA breaks and CIN promoting CMS3 and adenoma-carcinoma pathway characteristics. *C. jejuni*, *E. coli*, *H. ducreyi* and *H. hepaticus* produce CDT which causes cell cycle inhibition and double strand DNA breaks promoting characteristics of the adenoma-carcinoma pathway. *ETBF* promotes IL-8 secretion and promotes angiogenesis and cellular migration, characteristics observed in CMS2 CRC. *ETBF* increases WNT signalling and promotes EMT promoting adenoma-carcinoma pathway and CMS4 characteristics. Through BFT, *ETBF* promotes H_2_O_2_ production inducing double strand DNA breaks seen in tumours arising from the adenoma-carcinoma pathway. *C. perfringens* through CPE increases YAP activation promoting EMT as observed in CMS4 tumours. LPS, lipopolysaccharide; TME, tumour microenvironment; CIN, chromosomal instability; CDT, cytolethal distending toxin; *ETBF, enterotoxigenic Bacteroides fragilis*; CRC, colorectal cancer; BFT, *Bacteroides fragilis* toxin; CPE, *Clostridium perfringens* enterotoxin; EMT, epithelial mesenchymal transition. Image created with Biorender © 2022

CMS1, also referred to as MSI-immune, accounts for approximately 14% of CRC and is associated with MSI and CIMP phenotypes, *BRAF* mutations and the serrated pathway of carcinogenesis [23]. CMS1 tumours have high levels of immune-cell infiltration and activation. Patients with CMS1 tumours have the highest mortality rate after relapse of the four subtypes [23]. A study of the tumour microbiome in the context of CMS found that CMS1 cancers were significantly associated with tumour-resident oral pathogens, including *Fusobacterium* species, compared to other CMS subtypes [24]. Flanagan et al. had previously reported that *F. nucleatum* was significantly associated with CRC, when comparing healthy mucosa to CRC tumour tissue (a 45-fold increase of *F. nucleatum).* An increased abundance of *F. nucleatum* was also reported in precancerous adenomas compared to matched normal tissue, with bacterial load increasing as the degree of dysplasia increased [25]. Prognosis was negatively associated with *F. nucleatum*, and high abundance of tumour *F. nucleatum* was associated with a 2-year median survival rate compared to a survival rate greater than 3 years in tumours with low abundance [26]. These studies suggest that *Fusobacterium* species, particularly *F. nucleatum*, may play a role in colorectal carcinogenesis, particularly in CMS1 lesions. *Fusobacterium* species have a close relationship with immune response within the tumour microenvironment. Studies have shown that *F. nucleatum* drives immune-cell infiltration of CD11b + myeloid cells (Fig. 2), which differentiate into macrophages, granulocytes and dendritic cells, potentiating tumour promotion and immune suppression within the tumour microenvironment [27]. A multi-centre study reported that *F. nucleatum* was also associated with decreased intratumoural CD3 + T cell density, which may contribute to the immune suppressive tumour microenvironment associated with *Fusobacterium* species [28]. More recently, Mouradov et al. discussed a novel subtyping of oncomicrobial communities (OCS) [91]. The OCS1 subtype is associated with CMS1 and includes oral pathogens such as *F. nucleatum and F. periodonticum* [91]. Like CMS1 tumours, OCS1-associated tumours are more often right sided and predominantly MSI + , CIMP + and harbour *BRAF* mutations, compared to the OCS2 and OCS3 subtypes [91]. There were few prognostic associations observed using OCS, although MSS and left-sided tumours that had OCS1 were associated with a poorer prognosis, compared with tumours harbouring OCS2 and OCS3 [91].

### Consensus molecular subtype 2

CMS2, also referred to as the canonical subtype, accounts for ~ 37% of CRC cases and is characterised by tumours with high levels of somatic copy number alterations (SCNA) and chromosomal instability (CIN), which is a hallmark of cancers arising from the adenoma-carcinoma pathway [23, 29]. This subtype is also associated with increased WNT and MYC activation, leading to increased cellular proliferation [30]. Although a low bacterial biomass has been associated with CMS2 tumours overall, an increased abundance of both *Selenomonas* and *Prevotella* species was reported in a small study of 32 patients [24], [31]. The highest proportion of Bacteroidetes species was found in CMS2 tumours compared CMS1,3 and 4, with *Bacteroides fragilis* accounting for more than 50% of identified bacteria [24]. Some strains of Enterotoxigenic *B. fragilis* (ETBF) produce a toxin (BFT) that has been implicated in the development of CRC. ETBF is significantly increased in both cancerous and precancerous lesions compared to healthy mucosa, suggesting that ETBF may be a possible “driver bacterium” in CRC. [32, 33]. A study published in 2017 identified a positive correlation between the presence of ETBF and tubular adenomas, serrated polyps and low-grade dysplasia; however, no statistically significant association was found between ETBF and colorectal tumours [34]. A more recent study has shown that expression of the IL-8 gene (*CXCL8*) and protein is increased fivefold in CRC cell lines that had been co-cultured with ETBF [35]. IL-8 upregulation is known to promote colorectal carcinogenesis through increasing cellular proliferation, angiogenesis and cell migration (Fig. 2) [36].

### Consensus molecular subtype 3

CMS3, also referred to as the metabolic subtype, accounts for approximately 13% of all CRC, and tumours tend to express a mixed MSI phenotype and include both low SCNA and CIMP tumours [23]. CMS3 is characterised by *KRAS* mutations and metabolic deregulation [23]. There is very little data regarding the bacterial profile of CMS3 cancers. Purcell et al. found that CMS3 cancer subtype had the lowest abundance of *Bacteroides* and *Fusobacteria* species compared to both CMS1 and CMS2 cancers, but the overall tumour-resident bacterial biomass has also been reported to be low in this subtype [31]. A recent study found a high abundance of *Fusobacterium* species in CMS3 and CMS1 tumour tissue compared to CMS2 and CMS4 tissues based on data from The Cancer Genome Atlas (TCGA) and the European Genome-Phenome Archive (EGA) [37]. Formate, an oncometabolite produced by *F. nucleatum* and other bacteria, was found to stimulate the AhR signalling pathway and may influence cancer stem cell proliferation and migration, as well as xenobiotic metabolism (Fig. 2), which may push these tumours towards the CMS3 subtype, which is associated with metabolic dysregulation [23, 37, 38].

### Consensus molecular subtype 4

The final CMS subgroup, CMS4, is also known as the mesenchymal subtype and typically accounts for 23% of colorectal tumours. As with CMS2, CMS4 is characterised by high SCNA, indicative of CIN. CMS4 tumours present with stromal infiltration, TGF-β activation and increased angiogenesis and have the worst mortality rate of all CMS subtypes [23]. Whilst there is very little published data investigating bacterial interactions within CMS4 tumours, a recent study showed these tumours were differentially enriched with *Porphyromonas asaccharolytica* compared to CMS1 tumours [31]. These associations between the microbiome and CMS subtypes of CRC are summarised in Table 1.

**Table 1:** Associations and functional links between the microbiome and consensus molecular subtype pathways of colorectal carcinogenesis

Pathway	Bacteria	Mechanism
CMS1	*Fusobacterium nucleatum* [23, 31, 68, 71] *Enterotoxigenic Bacteroides fragilis (ETBF) *[95]	Intratumoral immune modulation. Increased myeloid cell infiltration [68] Lipopolysaccharide (LPS) binds to TLR4 resulting in increased M2 macrophage polarisation and promotes a proinflammatory tumour microenvironment [23, 31, 71] *ETBF associated with DNA hypermethylation in BRAFV600E* mice [95] *ETBF* associated with increased Th1 immune signatures and myeloid cell infiltration [95]
CMS2	*Enterotoxigenic Bacteroides fragilis* [53, 54]	Increased IL-8 expression, promotes carcinogenesis, angiogenesis and cellular migration [53, 54]
CMS3	*Fusobacterium spp*. (including *F. nucleatum*) [23, 37, 38]	Formate stimulates AhR signalling to induce cancer stem cell proliferation and migration. Induces xenobiotic metabolism [23, 37, 38]
	B2 E. coli + *pks* [45, 46]	Induces double strand DNA breaks and chromosomal instability [45, 46]
	*Clostridium perfringens* [59, 60]	*C. perfringens* toxin (CPE) induces YAP activation increasing cellular proliferation and survival [59, 60]
CMS4	*Porphyromonas asaccharolytica* [31, 77]	LPS attenuated immune modulatory effects of other LPS molecules contributing to immune evasive characteristics [31, 77]
	*Enterotoxigenic Bacteroides fragilis* [30, 36, 52–56]	BFT induces WNT signalling through nuclear accumulation of b-catenin to promote mesenchymal phenotype and cellular proliferation [36, 30, 52–56]
	*Clostridium perfringens* [59, 60]	CPE increased cytoplasmic expression of CLDN4, activation of YAP and induction of epithelial mesenchymal transition [59, 60]

## Bacterial associations with the adenoma-carcinoma pathway of colorectal cancer development

Cancers arising from the more common adenoma-carcinoma pathway are often associated with chromosomal instability. This includes changes in chromosomal structure and numbers, and may be detected as double-stranded DNA breaks. Several bacterial species can induce chromosomal instability via different mechanisms (Table 2).

**Table 2:** Associations and functional links between the microbiome and the adenoma-carcinoma and serrated pathways of colorectal carcinogenesis

Pathway	Bacteria	Mechanism
Adenoma-carcinoma	*Escherichia coli* [39, 42], *Helicobacter ducreyi* [42, 43], *Helicobacter hepaticus* [40, 41], *Campylobacter jejuni* [39, 44]	Cytolethal distending toxin (CDT) cell cycle inhibition inducing double strand DNA breaks [40–44]
	*B2 E.coli* + *pks* [45–47]	Colibactin induces double strand DNA breaks [45–47]
	Enterotoxigenic *Bacteroides fragilis* [48, 30, 49–56]	Bacteroides fragilis toxin (BFT) induces DNA damage though H_2_O_2._[48–50]. Increases WNT signalling pathways and promotes epithelial mesenchymal transition [36, 30, 52–56]
Serrated	*Clostridium perfringens* [24, 57–59]	*C. perfringens* enterotoxin increased cytoplasmic CLDN4, increasing YAP activation and pushing epithelial mesenchymal transition in cells expressing the *BRAF V600E* mutation [57–59]

### Cytolethal distending toxin

A range of gram-negative intestinal bacteria, including *E. coli*, and some *Campylobacter* and *Helicobacter* species produce cytolethal distending toxin (CDT) [39]. CDT is an AB_2_-type toxin, termed genotoxin, that can inhibit the cell cycle at G2, S and M phases and induce double-strand DNA breaks (Fig. 2) [40]. Double-strand DNA breaks lead to chromosomal instability and can be detected using the marker g-H2AX [41]. These double-strand DNA breaks have been observed in daughter cells of cell lines treated with CDT from both *E. coli* and *Helicobacter ducreyi*, suggesting that chromosomal instability is sustained during mitosis [42]. Recombinant CDT toxin from *Helicobacter hepaticus* could induce double-strand DNA breaks in human intestinal epithelial cell lines [41] and recombinant CDT from *H. ducreyi* increased double strand DNA breaks and chromosomal aberrations four-fold in human HCT116 colorectal cancer cell lines [43]. g-H2AX expression increased over 48 h, reaching a peak that coincided with cell cycle arrest at G_2_/M phase [41]. Further in vivo studies showed that treatment of mice with *H. hepaticus* CDT enhanced double-strand DNA breaks compared to an inactivated CDT, with a significant increase in g-H2AX expression (40). These studies suggest that CDT augments the ability of *H. hepaticus* to induce chromosomal instability, resulting in development of neoplasia through the conventional adenoma-carcinoma pathway. A more recent study has described how CDT from *Campylobacter jejuni* induces double-strand DNA breaks in both intestinal cell lines and enteroid models (Fig. 2) [44]. The *cdtB* subunit of CDT is essential for *C. jejuni* to induce carcinogenesis via the adenoma-carcinoma pathway; *APC*^*Min/*+^ mice that were colonised via oral gavage with wild-type *C. jejuni* showed a significantly higher tumour burden than those colonised with *C. jejuni mutcdtB* [44].

### Colibactin

Colibactin is a genotoxic metabolite, also capable of causing double-stranded DNA breakage, and is produced by several different bacteria. It is most well documented in B2 *E. coli* strains, where it is encoded by the *pks* island motif [45, 46]. In 2010, Cuevas-Ramos et al. used mouse colon loop models to show that *pks* + *E. coli* rather than *pks − E. coli* contributed to increased γH2AX expression, suggesting increased chromosomal instability (Fig. 2). Epithelial cancer cell lines treated with *pks* + *E. coli* showed atypical chromosome numbers, indicative of chromosomal instability [46]. Organoids derived from human colonic tissue that were exposed to *pks* + *E. coli* long-term expressed specific mutational signatures. These organoids showed an increase in single base substitutions (SBS-pks), specifically T > N, and a small indel signature, which was a single T deletion (ID-pks) [47]. Further analysis showed that both single-base substitutions and small indel signature (ID-pks) were associated with colorectal metastases compared to other cancers. A recent study of CRC has identified the *APC* oncogene to contain the highest number of *pks* motifs, whilst the *KRAS* gene, also characteristic of the conventional adenoma-carcinoma pathway and CMS3 subtypes, contained the fourth highest number of *pks* motifs [47]. Interestingly, the *BRAF* gene, commonly expressed in cancers arising from the sessile serrated pathway and CMS1 subtypes contained no *pks* motifs, further supporting the hypothesis that *pks* + *E.coli*, may contribute to adenoma-carcinoma pathway CRC development via the action of colibactin.

### *Bacteroidesfragilis* toxin (BFT)

Aside from genotoxins, such as CDT and colibactin, various bacteria can produce toxins that promote carcinogenesis through an array of mechanisms. As mentioned earlier, there are significant associations between ETBF and colorectal carcinogenesis, with several studies identifying increased ETBF abundance in the mucosa of CRC patients [32, 34] and in the stool samples of CRC patients compared to control populations [48]. ETBF produces the *B. fragilis* toxin (BFT), which is a metalloprotease, known to induce inflammation and increase epithelial permeability through its effect on E-cadherin [49]. Recombinant BFT was shown to induce DNA damage in colonic epithelial cell lines through spermine oxidase, by producing H_2_O_2_ through polyamine catabolism [50]. Whilst reactive oxygen species such as H_2_O_2_ have previously been implicated in inflammation and colorectal carcinogenesis, this is the only study suggesting how BFT may promote DNA damage, which leads to chromosomal instability, a feature of CMS2 and CMS4 colorectal tumours.

In addition to the genotoxic effect of BFT, potentially resulting in chromosomal instability, BFT may exert effects through other carcinogenic mechanisms. In vitro studies using CRC cell lines have demonstrated that enterotoxigenic *B. Fragilis* causes a decrease in E-cadherin expression through the action of BFT [35, 51]. BFT through an, as yet, unidentified receptor causes the cleavage of E-cadherin leading to b-catenin accumulation in the nucleus [51]. The nuclear accumulation of ß-catenin induces activation of *Wnt* signalling, a characteristic of the CMS2 subtype increasing cellular proliferation [30] and promotes epithelial-mesenchymal transition (Fig. 2) [52]. BFT also induces IL-8 secretion in CRC cell lines, resulting in downstream NF-κB activation [53]. Stat3 may play a crucial role in BFT-mediated IL-8 secretion, as induction of IL-8 secretion in response to ETBF treatment was diminished when CRC cell lines were treated with either a Stat3 or β-catenin antagonist. Whilst IL-8 can promote nonspecific drivers of carcinogenesis, such as cellular proliferation and inflammation [54], it is also a potent stimulator of angiogenesis and epithelial-mesenchymal transition (EMT) (Fig. 2) [36, 55]. Whilst their precursor lesions generally have a serrated appearance, CMS4 colorectal tumours typically express the CIN phenotype, and are characterised by high SCNA, increased epithelial–mesenchymal transition (EMT) and a high level of angiogenesis [23]. Loss of E-cadherin can lead to epithelial to mesenchymal transition [56]. ETBF may be involved in driving the CMS4 tumour phenotype development through disruption of E-cadherin and subsequent EMT, as well as inducing secretion of proangiogenic IL-8.

ETBF has also been shown to promote CMS1-like characteristics in *BRAF V600E* murine tumours, when exposed to long-term colonisation [95]. ETBF was associated with increased DNA hypermethylation compared to control mice [95]. The authors also found that tumours of *BRAF V600E* mice were enriched with Th1 immune signatures, including increased IFN-γ and IFN-α gene sets and increased myeloid-cell enrichment compared to controls [95].

## Bacterial associations with the serrated pathway of colorectal cancer development

### *Clostridiumperfringens*

*Clostridium perfringens* is often reported in CRC patients with bacteraemia and found both systemically and within the tumour [57, 58]. It has been reported in CMS 1–3 colorectal tumours, with CMS3 being the most enriched [24]. *C. perfringens* enterotoxin (CPE) has been shown to induce the activation of the yes-associated protein (YAP), which is involved in cellular proliferation, survival and cancer development (Fig. 2) [59]. Low-dose treatment of rat colonic epithelial cells with CPE showed an increase in cytoplasmic expression of the Claudin 4 (CLDN4) protein and decreased membranous CLDN4 expression (59). CLDN4 is an integral part of tight junctions; CPE binds to CLDN4 at the C-terminus leading to internalization of CLDN4 causing disruption of tight junctions, activation of YAP and subsequent epithelial–mesenchymal transition [60]. HT29 colorectal cancer cells expressing the *BRAF V600E* mutation showed an increased activation of YAP and cytosolic CLDN4 whilst a decrease in membranous CLDN4, compared to HCT116 *BRAF* wild-type cells, which showed no YAP activation or changes in CLDN4 when treated with CPE for 24 h. Activation of YAP by CPE promotes the development of serrated polyps and is associated with *BRAF* mutations [59]. This suggests that CPE potentiates epithelial-mesenchymal transition in CRC (Fig. 2) and may promote CRC development through the serrated pathway, potentially contributing to the development of CMS1 and CMS4 subtypes of CRC.

## The role of the microbiome in immune modulation

The interactions between the tumour and the immune cell populations within the TME play a pivotal role in tumorigenesis and development. It is now well established that the immune system can play dual roles in cancer, either inhibiting or enhancing tumour development, depending on the balance of immunomodulatory signals within the TME. The tumour microbiome can both directly and indirectly provide immunomodulatory signals and therefore alter the nature and magnitude of the anti-tumour response.

The immune system is broadly comprised of two inter connected arms—the innate and adaptive immune systems. Cells of the innate immune system recognise microbial pathogens through expression of pattern recognition receptors (PRRs), such as the toll-like receptors (TLRs). The PRRs recognise conserved microbial molecules, termed pathogen-associated molecular patterns (PAMPs). This recognition then activates signalling pathways that regulate multiple cellular functions, including cytokine release and adhesion molecule expression. This may, in turn, alter the differentiation and function of other cellular components, including regulatory cell populations, e.g. dendritic cells that control the adaptive immune response. In some settings, engagement of PRR by bacterial components results in a pro-tumorigenic environment, e.g. *F. nucleatum,* through TLR4 activation, has been shown to mediate M2 polarisation, promoting a proinflammatory, pro-tumour microenvironment [71]. Both *F. nucleatum* and *ETBF* can also induce infiltration of myeloid cells into the tumour microenvironment, promoting an immunosuppressive, pro-tumourigenic microenvironment [68, 69], with M2 tumour-associated macrophages associated with poor prognosis [70].

Bacterial lipopolysaccharide (LPS) and flagellin are examples of PAMPs. LPS is a cell wall component of gram-negative bacteria, aiding in structural integrity [63], and is implicated in different disease states through its inflammatory properties. Classically, *E. coli* LPS binds to TLR4 on the cell surface with the aid of LPS binding protein (LPB) and CD14. This then induces a signalling cascade, resulting in the activation of NF-kB, proinflammatory cytokines and interferons [64]. However, the structure of the individual LPS molecules can vary depending on the bacteria that they originate from, therefore altering the immune response to such pathogens.

LPS from *Porphyromonas gingivalis* contains unique long branched fatty acid chains in the biologically active lipid A portion of the molecule that differ from the shorter chains observed in *E. coli* LPS. As a result, *P. gingivalis* LPS, unlike *E. coli* LPS, does not require TLR4 for signalling and has been shown to signal through TLR2 [84, 85].

Several studies have shown that LPS from different bacterial species can either induce or suppress cytokine secretion, which alters immune response [31]. LPS from both *E.coli* and *Fusobacterium periodontium* appear to induce the release of several cytokines in animal models and peripheral blood mononuclear cell models, including IL-12, IFN-γ, IL-6 [73], IL-18, IL-1b [74], IL12-p70 [75], and IL-10 [76], which is consistent with a Th1 immune response [31, 84]. Sulit et al. showed an abundance of *Fusobacterium* species*.* in CMS1 tumours, and this may represent a possible mechanism of immune infiltration through initiation of a Th1 immune response in CMS1 colorectal cancer subtypes. In contrast, *P. gingivalis* LPS, which is structurally different from both *E.coli* and *F. periodonticum* LPS, induces IL-13, IL-5, and IL-10 cytokine production, suggesting a Th2 immune response [84]. A Th2 dominated immune response has been associated with an immunosuppressive tumour microenvironment, suggesting that *P. gingivalis* LPS may be involved in immunosuppression, allowing the tumour to progress [86]. Additionally, CMS4 tumours were found to be enriched with *P. asaccharolytica* and LPS related genes [31]. *P. asaccharolytica* LPS decreased IFN-γ secretion, as well as a range of other cytokines, including IL-6, and IL-1β, in peripheral blood mononuclear cells; this decrease in proinflammatory cytokines may contribute to the increased immunosuppression observed in CMS4 colorectal tumours [90].

Flagella are bacterial motility proteins that are recognised by the innate immune system via TLR5. They induce MyD88 activation, resulting in proinflammatory chemokine and cytokine release [65]. This in turn results in recruitment and activation of various immune cells, including dendritic cells, neutrophils and monocytes, initiating a feed-forward loop that induces further inflammatory cytokine production [66]. Dendritic cells can then activate the adaptive immune system resulting in recruitment of B and T cells and ultimately the neutralization and elimination of pathogens within the tumour microenvironment [67]. An increase in flagellar related assembly genes has been associated with faecal samples from CRC patients [61]. These samples from CRC patients were found to have an abundance of flagella-forming *Clostridium* species [61]. *Clostridium difficile* is found in abundance in colorectal cancer samples compared to adjacent healthy mucosa [62]. Through TLR5 signalling, *C. difficile* indices MAPK and NF-κB activation and the secretion of pro-inflammatory cytokines including IL-6 and IL-1β in mouse models [72]. It appears that flagella may contribute to a pro-inflammatory tumour microenvironment and an increased immune infiltration, similar to that observed in CMS1 tumours. Therefore, flagella may play a functional role in colorectal carcinogenesis towards a CMS1 subtype.

Further evidence of the ability of the microbiome to modulate immune responses has been provided by studies on the association between a patient’s microbiome and their response to immune checkpoint inhibitors (ICI). The administration of ICI can allow the development of effective anti-tumour responses in some patients but it is clear that other, poorly understood immunomodulatory factors modulate the response to these inhibitors. A study of CRC reported that patients who responded positively to PD1/PDL1 immunotherapy had 100% enrichment of *F. nucleatum,* compared to the non-responders who had a 47% enrichment [87]. Murine models administered *F. nucleatum* and an anti-PDL1 monoclonal antibody showed significant decrease in tumour growth, whilst *E. coli* and saline controls treated with an anti-PDL1 monoclonal antibody showed no changes in tumour growth [87]. The observed increase in efficacy may be due to *F. nucleatum* stimulating PDL1 expression through the STING pathway by the upregulation of cyclic GMP-AMP synthase (cGAS) and phosphorylation of STING [87]. Conversely, Jiang et al. identified a high abundance of *F. nucleatum* in non-responders to anti-PD-1 immunotherapy by looking at several cohorts of patients with CRC [94], To further understand the mechanism, murine models were subcutaneously implanted with tumour cells [94]. A decrease in anti-PD-1 efficacy was observed in mice treated with *F. nucleatum* and anti-PD-1 compared to anti-PD-1 alone [94]. There was also a decrease in CD8 + T cell infiltration into tumours of mice treated with *F. nucleatum* compared to untreated animals. Succinic acid, a metabolite of *F. nucleatum* appears to diminish the effect of immunotherapy [94]. Treatment of mice with either *F. nucleatum* or succinic acid resulted in decreased expression of IFN-β through the cGAS-STING pathway [94]. The studies from Gao et al. and Jaing et al. have demonstrated opposing effects of *F. nucleatum* and its involvement in immunotherapy response, suggesting that there may be other factors to consider outside of a single bacterium and its immediate effects, when understanding immune modulation. Immune modulation of intratumoural CD8 + T cells was also altered in mouse colon cancer models when colonised with either *Bifidobacterium pseudolongum*, *lactobacillus* or *Olsenella* species, compared to germ-free controls; an improvement in the efficacy of CTLA4 targeted immunotherapy was seen, with a significant decrease in both tumour size and weight compared to germ-free controls [88]. The efficacy of PDL1 targeted immunotherapy was also increased when mice were colonised with *B. pseudolongum,* but not to the same extend as observed for CTLA4 therapy [88]. *PKS* + *E. coli* has already been discussed for its role in CIN in the adenoma-carcinoma pathway. In addition, recent evidence has shown that *PKS* + *E. coli* is sufficient to inhibit response to anti-PD1 immunotherapy in murine MC38 tumour graft models [89]. *PKS* + *E. coli* was associated with a significant decrease in total CD3 + and CD8 + T cells in both mucosa and tumours in tumour-graft mouse models treated with *PKS* + *E. coli*, compared to untreated controls. More recently, *Proteus mirabilis,* which is typically associated with intestinal inflammation [92], has been shown to improve the efficacy of anti-PD-L1 immunotherapy in mice [93]. *P. mirabilis*, expressing the *FlaB* gene, could localise to the tumour site following intravenous administration [93]. Tumours of mice treated with *FlaB* + *P. mirabilis* showed an increased infiltration of cytotoxic T cells compared to untreated mice, The expression of PD-L1 on the infiltrating immune cells when mice were treated with *FlaB* + *P. mirabilis* suggests a possible mechanism to improve immunotherapy efficacy [93]. *BRAFV600E* mice that have had long-term exposure to *ETBF* also showed an improved response rate to anti-PD-L1 immunotherapy [95]. *ETBF-*treated mice showed a reduction in tumour counts per mouse and per colonic region compared to control mice suggesting *ETBF* attenuates anti-PDL1 immunotherapy in *BRAFV600E* mice [95]. These studies suggest that specific bacteria present in the tumour microenvironment can modulate the immune system’s response to immune checkpoint inhibitors and thereby influence the course of cancer progression.

## Systemic effects of gut microbiome

Whilst this review focused on the role of the tumour microbiome and local effects within the colorectal tumour microenvironment, it is important to mention the systemic effects of the gut microbiome. As previously mentioned, bacteria such as ETBF, through its BFT toxin, causes increased intestinal barrier permeability [78]. This, in turn may allow bacteria and bacterial components to pass through the intestinal wall and into the bloodstream. When LPS, a component of gram-negative bacteria, enters the bloodstream, it can activate an inflammatory cascade by binding to TLR4 on immune cells, stimulating the release of proinflammatory cytokines and chemokines [79], which may act systemically upon the tumour microenvironment.

Conversely, short-chain fatty acids (SCFAs), including butyrate and propionate, are produced through bacterial metabolism and are known to improve intestinal barrier function through upregulation of tight-junction components [80]. The ability of SCFAs to modulate immune cells has also been demonstrated. Using mouse models, Arpaia et al. demonstrated that both butyrate and propionate promoted the generation of anti-inflammatory Treg cells [81]. Whilst the production of Treg cells suppresses systemic inflammation, an increased Treg infiltration within the TME is often associated with a poor prognosis, due to their ability to suppress anti-tumour immunity (82).

## Conclusion

The tumour microenvironment is a complex, heterogeneous landscape that greatly impacts the progression of CRC. This review has described several bacterial mechanisms that may drive cancer development along specific pathways and in CMS subtypes, through interactions with specific aspects of the tumour microenvironment. The tumour microbiome can influence cancer progression through genomic alterations, increased cellular proliferation and migration, as well as modulation of both innate and adaptive immune responses. The microbial landscape within each tumour subtype is unique and diverse, and whilst this review has discussed individual mechanisms by which bacteria may influence the CRC subtypes and progression, further work is needed to better understand the crosstalk between bacterial communities and the immune, metabolic and genomic landscape of individual tumours. However, it is clear that the tumour microbiome contributes significantly to colorectal carcinogenesis.

## Data Availability

No datasets were generated or analysed during the current study.
